# Cardiac and Renal SARS-CoV-2 Viral Entry Protein Regulation by Androgens and Diet: Implications for Polycystic Ovary Syndrome and COVID-19

**DOI:** 10.3390/ijms22189746

**Published:** 2021-09-09

**Authors:** Samar Rezq, Alexandra M. Huffman, Jelina Basnet, Licy L. Yanes Cardozo, Damian G. Romero

**Affiliations:** 1 Department of Cell and Molecular Biology, University of Mississippi Medical Center, 2500 N, State Street, Jackson, MS 39216, USA; srezq@umc.edu (S.R.); ahuffman@umc.edu (A.M.H.); jbasnet@umc.edu (J.B.); lyanes@umc.edu (L.L.Y.C.); 2 Mississippi Center of Excellence in Perinatal Research, University of Mississippi Medical Center, 2500 N, State Street, Jackson, MS 39216, USA; 3 Women’s Health Research Center, University of Mississippi Medical Center, 2500 N, State Street, Jackson, MS 39216, USA; 4 Cardio Renal Research Center, University of Mississippi Medical Center, 2500 N, State Street, Jackson, MS 39216, USA; 5 Department of Pharmacology and Toxicology, Faculty of Pharmacy, Zagazig University, Zagazig 44519, Egypt; 6 Department of Medicine, University of Mississippi Medical Center, 2500 N, State Street, Jackson, MS 39216, USA

**Keywords:** polycystic ovary syndrome, COVID-19, SARS-CoV-2, angiotensin converting enzyme 2, androgens, obesity

## Abstract

The susceptibility and the severity of coronavirus disease 2019 (COVID-19) caused by severe acute respiratory syndrome coronavirus 2 (SARS-CoV-2) are associated with hyperandrogenism, obesity, and preexisting pulmonary, metabolic, renal, and cardiac conditions. Polycystic ovary syndrome (PCOS), the most common endocrine disorder in premenopausal women, is associated with obesity, hyperandrogenism, and cardiometabolic dysregulations. We analyzed cardiac, renal, circulatory, and urinary SARS-CoV-2 viral entry proteins (ACE2, TMPRSS2, TMPRSS4, furin, cathepsin L, and ADAM17) and androgen receptor (AR) expression, in a peripubertal androgen exposure model of PCOS. Peripubertal female mice were treated with dihydrotestosterone (DHT) and low (LFD) or high (HFD) fat diet for 90 days. HFD exacerbated DHT-induced increase in body weight, fat mass, and cardiac and renal hypertrophy. In the heart, DHT upregulated AR protein in both LFD and HFD, ACE2 in HFD, and ADAM17 in LFD. In the kidney, AR protein expression was upregulated by both DHT and HFD. Moreover, ACE2 and ADAM17 were upregulated by DHT in both diets. Renal TMPRSS2, furin, and cathepsin L were upregulated by DHT and differentially modulated by the diet. DHT upregulated urinary ACE2 in both diets, while neither treatment modified serum ACE2. Renal AR mRNA expression positively correlated with Ace2, Tmprss2, furin, cathepsin L, and ADAM17. Our findings suggest that women with PCOS could be a population with a high risk of COVID-19-associated cardiac and renal complications. Furthermore, our study suggests that weight loss by lifestyle modifications (i.e., diet) could potentially mitigate COVID-19-associated deleterious cardiorenal outcomes in women with PCOS.

## 1. Introduction

The severe acute respiratory syndrome coronavirus 2 (SARS-CoV-2), a member of the coronavirus (CoV) family, causes Coronavirus Disease 2019 (COVID-19). The high transmissibility, morbidity, and mortality, as well as the unpredictability in outcomes differentiate COVID-19 from viruses of the same family [1,2]. The symptoms associated with COVID-19 range from fever to cough and shortness of breath which can rapidly progress to respiratory and cardiac failure and death [3]. Despite the high unpredictability of the final outcomes of the disease, some populations are at higher risk of severe COVID-19 outcomes, including the elderly, the immunocompromised, and those with preexisting pulmonary conditions or chronic obstructive pulmonary disease (COPD) [4]. Interestingly, the virus also targets a number of extrapulmonary organs; therefore, preexisting cardiometabolic comorbidities such as obesity, diabetes, hypertension, and renal disease are considered key risk factors for COVID-19 severity [5,6,7,8,9,10].

New clinical evidence suggests that men are both more susceptible than women and are at greater risk of experiencing a more severe form of COVID-19 independent of age [11,12]. This increased vulnerability could be explained by increased androgen levels [13,14], as well as sex differences in immune responses [15]. Polycystic ovary syndrome (PCOS) represents the most common endocrine disorder in reproductive age women, with a prevalence that ranges from 4–21% depending on the diagnostic criteria used [16]. The features that characterize PCOS are hyperandrogenism, oligo- or anovulation, and polycystic ovary morphology. Clinical or biochemical hyperandrogenism is present in more than 80% of women with PCOS [17]. In addition, women with PCOS have higher incidence of cardiovascular diseases, such as increased blood pressure [18], left ventricular hypertrophy [19], and renal injury [20]. Moreover, the majority of women with PCOS are obese or overweight [21] and display insulin resistance [22]. The high prevalence of multiple risk factors in PCOS that overlap with those of COVID-19 severity underscores women with PCOS as a critical patient population at potentially higher risk for adverse disease outcomes. Moreover, a recent epidemiological study suggests that women with PCOS have a remarkably increased risk for COVID-19 infection compared to control subjects [23]. Although the main concern in COVID-19 is the extensive alveolar damage and acute respiratory failure, there is a need to investigate the other organs involved [24]. COVID-19 has been shown to adversely affect the cardiovascular system in a large proportion of patients and to exacerbate preexisting cardiac injury, an effect that is evident even after COVID-19 acute phase recovery [25,26,27,28,29]. Additionally, patients with elevated cardiac injury markers are at a significantly increased risk of COVID-19 death [30]. Notably, approximately 5–7% of COVID-19 patients develop acute renal injury, and this rate was reported to be higher (11.9%) in patients with elevated baseline serum creatinine [5,31,32,33].

SARS-CoV-2 viral entry into the host cells requires the coordinated action of multiple host cell proteins. Angiotensin converting enzyme 2 (ACE2) is a host cell surface protein that binds to SARS-CoV-2 S-protein and acts as the SARS-CoV-2 receptor [34,35]. The SARS-CoV-2 S-protein requires priming by host cell proteases to facilitate viral fusion and entry. The transmembrane serine protease 2 (TMPRSS2) is the main protease that cleaves SARS-CoV-2 S-protein. However, there are other host proteases (furin, cathepsin L, and TMPRSS4) that have been suggested to prime SARS-CoV-2 S-protein in a tissue-specific manner. Moreover, the ACE2 sheddase ADAM17 can cleave ACE2 ectodomain from its membrane-bound state to its soluble form, effectively regulating SARS-CoV-2 receptor levels [36].

We previously reported that excess androgens in a rodent model of PCOS upregulate ACE2 protein expression in the heart, kidney, and small intestine [37]. Whether obesity, a comorbidity of both PCOS and COVID-19, modulates SARS-CoV-2 viral entry factors in cardiovascular and renal systems in PCOS is unknown. In the current report, we analyzed cardiac and renal SARS-CoV-2 viral entry proteins regulation by an obesogenic diet in a well-established mouse experimental model of PCOS that exhibits a breadth of endocrine, reproductive, and metabolic features that closely resemble the human pathology [38].

## 2. Results

### 2.1. HFD Exacerbates DHT-Mediated Obesity, and Cardiac and Renal Hypertrophy

**Figure 1:**
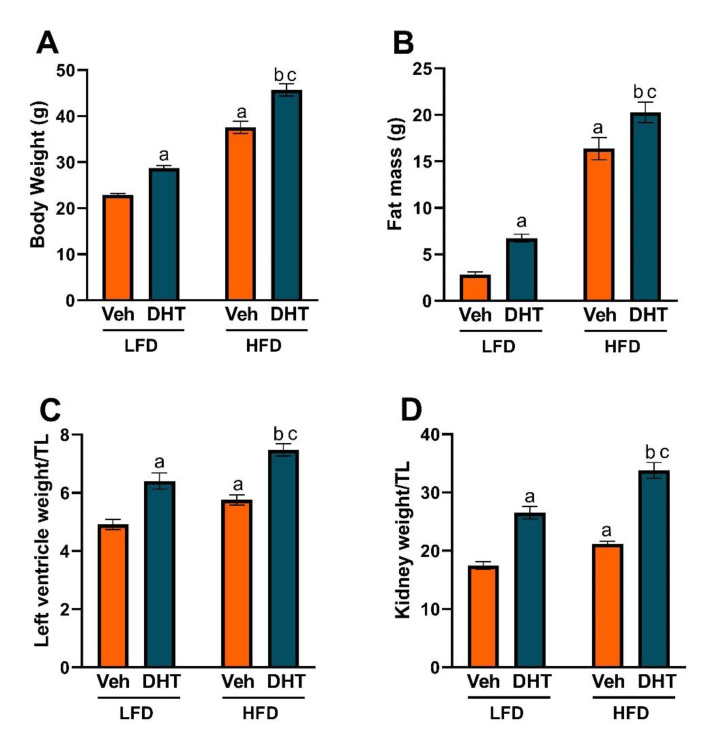
Effect of DHT and diet on body weight and composition, and cardiac and renal hypertrophy. Animals were treated with dihydrotestosterone (DHT) or vehicle (Veh) and maintained in low (LFD) or high (HFD) fat diet for 90 days. (**A**) Body weight was determined by gravimetry. (**B**) Fat mass was determined by EchoMRI. Left ventricle (LV) (**C**) and kidney (**D**) weights were determined by gravimetry and corrected by tibia length (TL). Data are expressed as mean ± SEM. *N* = 8/group. Data were analyzed by two-way ANOVA followed by Fisher’s LSD test. ^a^ *p* < 0.05 vs. LFD-Veh; ^b^ *p* < 0.05 vs. HFD-Veh; ^c^ *p* < 0.05 vs. LFD-DHT.

To analyze the effect of diet-induced obesity in the mouse model of PCOS by peripubertal androgen exposure, we studied body weight and composition, and cardiac and renal hypertrophy. After 90 days of DHT treatment, LFD-fed DHT-treated (LFD-DHT) mice had significantly increased body weight, fat mass, and left ventricle (LV), and kidney hypertrophy compared with LFD-fed vehicle-treated (LFD-Veh) mice (Figure 1A–D). Moreover, HFD-fed vehicle-treated (HFD-Veh) mice showed an increase in all the aforementioned parameters compared with LFD-Veh mice. Notably, HFD-fed DHT-treated (HFD-DHT) mice showed an exacerbation in body weight, fat mass, LV, and kidney hypertrophy compared with HFD-Veh and LFD-DHT.

### 2.2. Cardiac and Renal Androgen Receptor Expression Is Upregulated by DHT

**Figure 2:**
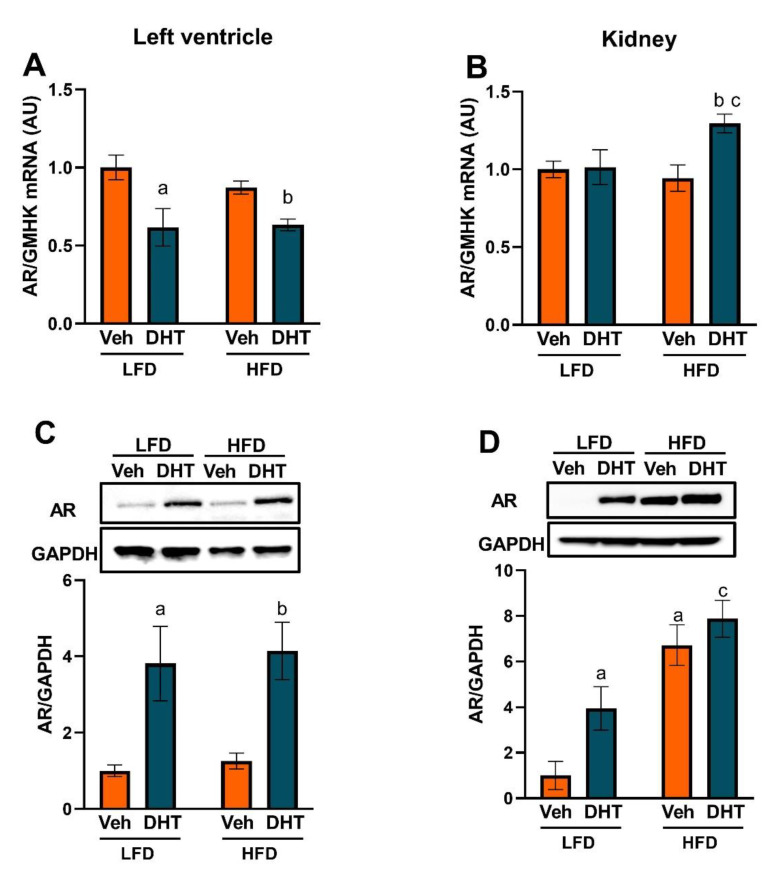
Effect of DHT and diet on androgen receptor mRNA and protein expression. Animals were treated with dihydrotestosterone (DHT) or vehicle (Veh) and maintained in low (LFD) or high (HFD) fat diet for 90 days. Left ventricle (LV) (**A**) and kidney (**B**) Androgen receptor (AR) mRNA was quantified by RT-qPCR (*N* = 6–8/group). LV (**C**) and kidney (**D**) AR protein was quantified by Western-blot (*N* = 4/group). Data are expressed as mean ± SEM. Data were analyzed by two-way ANOVA followed by Fisher’s LSD test. ^a^ *p* < 0.05 vs. LFD-Veh; ^b^ *p* < 0.05 vs. HFD-Veh; ^c^ *p* < 0.05 vs. LFD-DHT.

We then analyzed if the LV and kidneys express the androgen receptor (AR) protein and if the AR is subjected to regulation by androgens and/or an obesogenic diet. DHT treatment caused a minimal decrease in LV androgen receptor (AR) mRNA levels in both LFD and HFD (Figure 2A). However, DHT treatment caused a notable increase in LV AR protein expression in both LFD and HFD (Figure 2C). In the kidney, DHT treatment increased renal AR mRNA expression in HFD with no effect in LFD (Figure 2B). At the protein level, DHT increased renal AR protein expression in LFD (Figure 2D). Surprisingly, HFD by itself increased renal AR protein expression, an upregulation that was not additionally affected by DHT co-treatment (Figure 2D).

### 2.3. Cardiac and Renal Expression of ACE2 and Cellular Proteases Is Modulated by Both DHT and HFD

**Figure 3:**
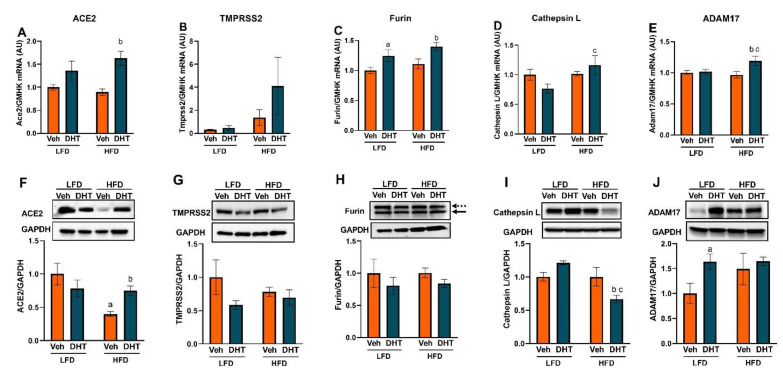
Effect of DHT and diet on cardiac SARS-CoV-2 viral entry proteins expression. Animals were treated with dihydrotestosterone (DHT) or vehicle (Veh) and maintained in low (LFD) or high (HFD) fat diet for 90 days. Ace2 (**A**), Tmprss2 (**B**), furin (**C**), cathepsin L (**D**), and ADAM17 (**E**) mRNA was quantified by RT-qPCR and standardized to the geometric mean of four housekeeping genes (HGMK) (*N* = 6/group). ACE2 (**F**), TMPRSS2 (**G**), furin (**H**), cathepsin L (**I**), and ADAM17 (**J**) protein was quantified by Western-blot and normalized to GAPDH (*N* = 4/group). Total furin (**H**) was quantified as the sum of profurin (dashed arrow) and cleaved soluble furin (solid arrow). Data are expressed as mean ± SEM. Data were analyzed by two-way ANOVA followed by Fisher’s LSD test. ^a^ *p* < 0.05 vs. LFD-Veh; ^b^ *p* < 0.05 vs. HFD-Veh; ^c^ *p* < 0.05 vs. LFD-DHT.

We then analyzed cardiac and renal SARS-CoV-2 viral entry protein regulation by androgens and an obesogenic diet at the mRNA and protein levels. In the LV, SARS-CoV-2 viral entry protein mRNA levels were not affected by DHT treatment in LFD with the exception of furin which was elevated (Figure 3A–E). However, DHT treatment significantly upregulated LV Ace2, furin, and ADAM17 mRNA expression in HFD-fed mice (Figure 3A–E). In alignment with the mRNA expression data, LV SARS-CoV-2 viral entry protein levels were not affected by DHT treatment in LFD-fed mice with the exception of ADAM17 which was upregulated compared with LFD-Veh mice (Figure 3F–J). At the protein level, LV ACE2 expression was decreased by HFD in control animals, but this decrease was abolished by DHT treatment (Figure 3F). Moreover, LV cathepsin L protein levels were decreased in HFD-DHT mice compared with LFD-DHT and HFD-Veh counterparts (Figure 3I). LV ADAM17 protein expression was upregulated by DHT exclusively in LFD-fed mice (Figure 3J). Tmprss4 mRNA and protein were undetectable in LV samples.

**Figure 4:**
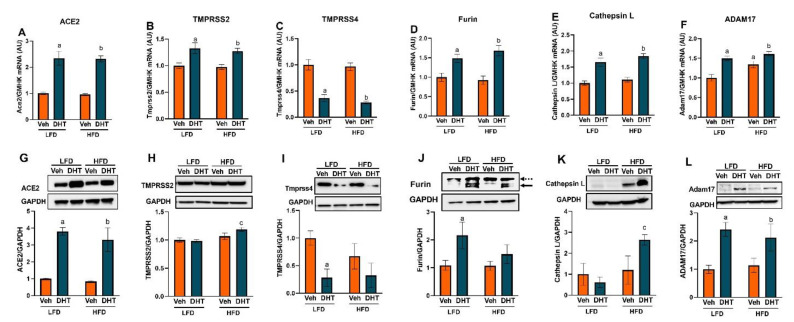
Effect of DHT and diet on renal SARS-CoV-2 viral entry proteins expression. Animals were treated with dihydrotestosterone (DHT) or vehicle (Veh) and maintained in low (LFD) or high (HFD) fat diet for 90 days. Ace2 (**A**), Tmprss2 (**B**), Tmprss4 (**C**), furin (**D**), cathepsin L (**E**), and ADAM17 (**F**) mRNA was quantified by RT-qPCR and standardized to the geometric mean of four housekeeping genes (HGMK) (*N* = 6–8/group). ACE2 (**G**), TMPRSS2 (**H**), TMPRSS4 (**I**), furin (**J**), cathepsin L (**K**), and ADAM17 (**L**) protein was quantified by Western-blot and normalized to GAPDH (*N* = 4/group). Total furin (**J**) was quantified as the sum of profurin (dashed arrow) and cleaved soluble furin (solid arrow). Data are expressed as mean ± SEM. Data were analyzed by two-way ANOVA followed by Fisher’s LSD test. ^a^ *p* < 0.05 vs. LFD-Veh; ^b^ *p* < 0.05 vs. LFD-Veh; ^c^ *p* < 0.05 vs. LFD-DHT.

In the kidney, SARS-CoV-2 viral entry proteins were significantly regulated at both the mRNA and protein levels (Figure 4A–L). At the mRNA level, Ace2 and the cellular proteases Tmprss2, furin, cathepsin L, and ADAM17 were upregulated by DHT in kidney of LFD- and HFD-fed mice (Figure 4A–F). On the other hand, renal Tmprss4 mRNA expression was downregulated in DHT-treated mice on both diets (Figure 4C). Notably, HFD by itself upregulated renal ADAM17 mRNA expression compared with LFD-Veh mice (Figure 4F). At the protein level, DHT treatment upregulated renal ACE2 and ADAM17 levels in mice maintained in both LFD and HFD (Figure 4G,L). On the other hand, TMPRSS2 and cathepsin L protein levels were only significantly upregulated by DHT in HFD-fed mice compared to their LFD-fed counterparts (Figure 4H,K). In parallel with mRNA determinations, renal TMPRSS4 expression was significantly downregulated in LFD-DHT mice and showed a tendency to decrease in HFD-DHT mice (Figure 4I). Renal total furin protein levels were increased by DHT exclusively in LFD-fed mice (Figure 4J). Moreover, soluble (active) renal furin protein was increased by DHT (Supplementary Figure S1).

### 2.4. DHT Increases Urinary ACE2 Levels

**Figure 5:**
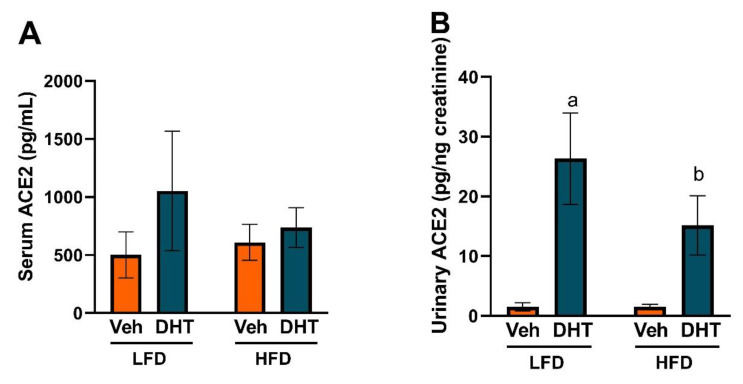
Effect of DHT and diet on serum and urinary ACE2. Animals were treated with dihydrotestosterone (DHT) or vehicle (Veh) and maintained in low (LFD) or high (HFD) fat diet for 90 days. Serum (**A**, *N* = 4–7/group) and urinary (**B**, *N* = 4–6/group) ACE2 were quantified by ELISA. Urinary ACE2 was corrected by urinary creatinine. Data are expressed as mean ± SEM. Data were analyzed by two-way ANOVA followed by Fisher’s LSD test. ^a^ *p* < 0.05 vs. LFD-Veh; ^b^ *p* < 0.05 vs. HFD-Veh.

Since renal ADAM17 protein was significantly upregulated by DHT, we analyzed if that regulation translated into a modulation of circulating and urinary soluble ACE2 levels. Serum ACE2 protein levels were similar among all groups (Figure 5A). However, DHT-treated mice showed a remarkable ~18- and ~10-fold increase in urinary ACE2 protein levels in LFD and HFD, respectively (Figure 5B).

### 2.5. Renal AR Expression Positively Correlates with Ace2, Tmprss2, Furin, Ctsl, and ADAM17 Expression

**Figure 6:**
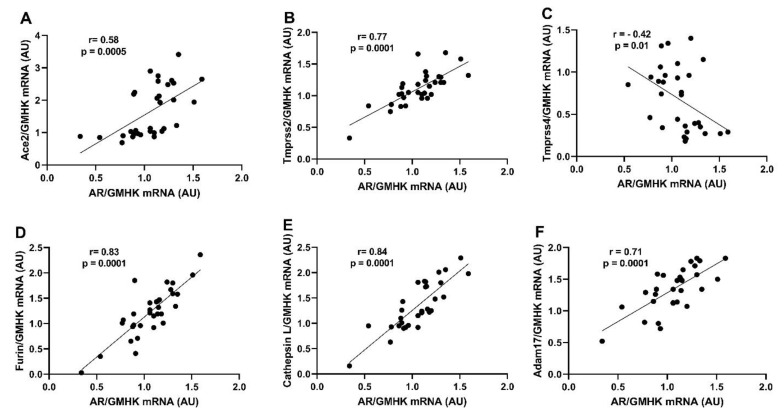
Correlations between renal androgen receptor mRNA expression and SARS-CoV-2 viral entry proteins. Animals were treated with dihydrotestosterone (DHT) or vehicle (Veh) and maintained in low (LFD) or high (HFD) fat diet for 90 days. Androgen receptor and SARS-CoV-2 viral entry proteins renal mRNA expression was quantified by RT-qPCR. Pearson’s correlation coefficients (r) were calculated between androgen receptor and Ace2 (**A**), Tmprss2 (**B**), Tmprss4 (**C**), furin (**D**), cathepsin L (**E**), and ADAM17 (**F**) mRNA expression.

Finally, we performed a correlation analysis between AR and SARS-CoV-2 viral entry proteins expression. AR mRNA expression showed a significant positive correlation with SARS-CoV2 receptor Ace2 and all the cellular proteases studied (Tmprss2, furin, cathepsin L, and ADAM17) with the exception of Tmprss4, which was negatively correlated with AR expression (Figure 6A–F). On the other hand, LV AR mRNA expression was not significantly correlated with Ace2 or any of the cellular proteases with the exception of furin, which showed a negative correlation with AR (*r* = −0.44, *p* = 0.03).

## 3. Discussion

Women with PCOS have increased prevalence of multiple cardiovascular risk factors including obesity [39]. Body mass index (BMI) is positively associated with a worsened cardiometabolic phenotype in women with PCOS [40]. Moreover, weight loss by lifestyle modifications or bariatric surgery improves the deleterious cardiometabolic profile associated with PCOS [41]. Women with PCOS are at increased risk for COVID-19 infection and higher incidence of COVID-19-associated symptoms [23,42]. In the current study, our findings suggest that women with PCOS could be a population at high potential risk of increased cardiac and renal SARS-CoV-2 infection and consequently the possibility of experiencing severe COVID-19-associated cardiac and renal deleterious outcomes. Moreover, our results highlight that an obesogenic diet differentially modulate SARS-CoV-2 viral entry proteins in cardiac and renal tissues.

The main findings of our study are: (1) HFD exacerbates DHT-induced increase in body weight, fat mass, and cardiac and renal hypertrophy; (2) DHT increases cardiac and renal AR expression; (3) Cardiac ACE2 and several cellular proteases (furin, cathepisn L, and ADAM17) are upregulated by DHT at the mRNA or protein levels; (4) Renal ACE2 and several cellular proteases (TMPRSS2, furin, cathepisn L, and ADAM17) are upregulated by DHT, both at the mRNA and protein levels; (5) An obesogenic diet differentially modulates multiple SARS-CoV-2 viral entry proteins in cardiac and renal tissues.

Obesity is a common metabolic disease, affecting 42.4% of US adults in the most recent estimates from the 2017–2018 period [43]. Obesity prevalence is similar between adult men and women overall or by age group [43]. Similar figures are observed worldwide with the World Health Organization reporting that 39% of adults aged 18 years and over (39% of men and 40% of women) were overweight in 2016, and 13% (11% of men and 15% of women) were obese. The main causes of obesity are an increased intake of energy-dense foods that are high in fat and sugars and a decrease in physical activity due to the increasingly sedentary nature of many forms of work, changing modes of transportation, and increasing urbanization. In the US, data from the National Health and Nutrition Examination Survey (NHANES) from the 1999–2016 cycles showed that despite some minimal improvements in total and low-quality carbohydrate intake, saturated fat intake still exceeded dietary guidelines [44]. Total fat intake increased over time, and saturated fat made up about 12% of daily calories, 2% higher than the recommended daily amount. Assessment of diet quality by the Healthy Eating Index 2015 (HEI-2015), which measures adherence to key recommendations in the 2015–2020 Dietary Guidelines for Americans in a 0–100 range (higher scores indicating better diet quality), showed a modest 2 points increase in the abovementioned period, from 55.7 to 57.7, still far from a satisfactory score. Based on these human data, obesity is highly prevalent worldwide, and inadequate fat intake could be one of the causes leading to this epidemic. Our studies modulating obesity in a model of PCOS using a HFD are aimed to address the effect of the obesity pandemic in women with PCOS.

Multiple studies have shown that COVID-19 disease progression severity and mortality are associated with underlying comorbidities including obesity, diabetes, and cardiovascular and renal disease, among others [5,6,7,8,9,10]. In particular, BMI is strongly positively associated with hospitalization, intensive care unit admission, invasive mechanical ventilation, and death in COVID-19 patients [9,45,46,47,48]. Obesity is highly prevalent in PCOS, as evidenced by a meta-analysis that included more than 35 studies and 15,000 women with PCOS; the study found that women with PCOS had increased prevalence of overweight, obesity, and abdominal obesity [21]. Central or abdominal obesity, assessed by waist circumference, is strongly positively associated with Homeostatic Model Assessment for Insulin Resistance (HOMA-IR), an index of insulin resistance, in women with PCOS [49]. In our study, HFD-DHT mice showed a ~1.5- and ~3.0-fold increase in body weight and fat mass compared with LFD-DHT mice (Figure 1), with the body weight having a synergistic interaction between DHT and HFD. These findings suggest a critical role of the diet in PCOS in body weight and composition in the presence of hyperandrogenism. The aforementioned findings by us and others strongly suggest a link between obesity, COVID-19, and PCOS that is worth further exploring at the molecular level and was the crux of the current study.

PCOS is diagnosed by the presence of at least two of the following three criteria: hyperandrogenism (biochemical or clinical), ovulatory dysfunction (oligo- or anovulation), and polycystic ovary morphology, following the Rotterdam criteria [50]. Recent reports suggest that women with PCOS are at increased risk of COVID-19 infection and worse outcomes. For example, a cross-sectional case-control study showed that COVID-19 positive outpatient women with PCOS had a higher incidence of COVID-19-associated symptoms such as low-grade fever, anosmia, ageusia, and dry cough, among others, when compared with COVID-19-positive non-hyperandrogenemic control women [42]. Moreover, a large epidemiological study of more than 20,000 women with PCOS, matched 1:4 with control women, found a 33% increase risk for COVID-19 infection compared to control women after adjusting for BMI, age, and impaired glucose regulation [23]. Those recent findings stress the need to expand our knowledge on the interaction between PCOS and COVID-19.

Death from COVID-19-associated cardiac injury occurs in ~20–30% of hospitalized patients and represents 40% of total deaths [27,28,51]. In a retrospective analysis of 5449 COVID-19 patients, 37% of them had acute kidney injury, which was more pronounced in patients with respiratory failure [52]. Importantly, the adverse effects of COVID-19 infection on the cardiovascular and renal systems are exacerbated in patients with preexisting cardiac or renal injury or dysfunction [5,28]. In our study, both DHT and HFD caused cardiac and renal hypertrophy (Figure 1). Surprisingly, the combination treatment of DHT and HFD produced a synergistic effect on both cardiac and renal hypertrophy that was larger than the effect of each individual intervention. Both cardiac hypertrophy and renal tubular injury were found to be clinical features of SARS-CoV-1 and -2 patients in post-mortem examination, despite presenting with normal kidney weights [53,54,55]. The cardiac and renal hypertrophy triggered by DHT or HFD treatment and their synergistic effect suggests a potential to aggravate COVID-19 associated pathology in those tissues in PCOS. Together, our findings and those from others suggest the likelihood of cardiorenal injury in clinical and experimental models of PCOS [19,56,57,58]. Those results highlight this patient population as being in a high-risk category of more severe cardiac and/or renal outcomes following COVID-19 infection, especially in those who are overweight or obese.

The increased case incidence and disease progression severity from COVID-19 observed in men [11,12] can be, at least partially, explained by increased androgen levels and/or AR activation [13,14]. This hypothesis is indirectly supported by the clinical evidence that shows that men with androgenetic alopecia experience more severe COVID-19 symptoms [13] and that pre-pubertal males are more resistant to infection compared to adults [14]. Biochemical elevation in circulating androgens, or hyperandrogenemia, is present in ~80% of women with PCOS [17]. Furthermore, the Androgen Excess and PCOS (AE-PCOS) Society considers PCOS a disorder of androgen excess or hyperandrogenism [59]. Moreover, the PCOS diagnostic criteria from the AE-PCOS Society, one of the three criteria used worldwide for PCOS diagnosis, considers hyperandrogenism (clinical and/or biochemical) a sine qua non condition for the syndrome diagnosis [60]. The main sources of androgens in PCOS are the ovary and the adrenal glands [61]. However, the adipose tissue, particularly the subcutaneous adipocytes, has been shown to generate testosterone from the precursor androstenedione by the enzymatic action of 17β-hydroxysteroid dehydrogenase type 5 (17β-HSD5) or AKR1C3 enzyme [62,63]. Notably, AKR1C3 expression is upregulated in the subcutaneous adipose tissue from women with PCOS [63]. Moreover, AKR1C3 expression is decreased in non-PCOS obese women following weight loss [62]. Those findings suggest that the adipose tissue is not only a target of androgens but also a source for those sex hormones in both PCOS and obesity. Additionally, those findings may explain the higher circulating androgens in normally menstruating obese women [64] and in women with atypical PCOS who have no evidence of adrenal and/or ovarian hyperandrogenism [65]. Furthermore, this is supported by studies that reported a positive correlation between androgens and BMI in PCOS [66] and between subcutaneous fat 17β-HSD5 expression and BMI in non-PCOS obese women [62]. Our findings showed that DHT-treated mice had increased renal and cardiac AR protein levels (Figure 2), suggesting that they exhibit increased androgen signaling due to the increase in both AR activation (due to increased ligand levels) and AR expression. Notably, HFD treatment alone increased renal AR protein expression, which may be attributed to an increase in adipose tissue-derived androgens due to an increase in fat mass that could lead to increased circulating androgens in HFD-fed mice. Interestingly, circulating androgens in women with PCOS are positively associated with the severity of metabolic dysfunction and obesity [25,26,27,28], both being well-recognized comorbidities in the severity of COVID-19 [29].

Coronaviruses’ viral entry into target cells depends on the binding of the S1 subunit of the spike (S) protein to a cellular receptor followed by S protein proteolytic cleavage and activation at the S1/S2 subunits interphase. The fusion of viral and cellular membranes is then driven by the S2 transmembrane subunit. ACE2 is the cellular receptor for SARS-CoV and SARS-CoV-2 [67]. A number of cellular proteases can proteolytically cleave, or prime, CoV S proteins. The main cellular protease identified is the transmembrane protease serine 2 (TMPRSS2). Other proteases such as TMPRSS4, cathepsin L, furin, and ADAM17 can also prime SARS-CoV S proteins [34,35,68,69]. Availability of these proteases on target cells largely determines whether CoVs enter cells through plasma membrane or endocytosis and which cells or tissues are susceptible to CoV infection.

Clinical studies have shown that high ACE2 protein expression in specific organs, including the heart and the kidney, positively correlates with the degree of organ damage in SARS patients [12]. ACE2 cleaves Ang II to form the vasodilatory and antiproliferative peptide Ang (1–7) [70]. ACE2 is highly expressed in the kidney and heart [71]. Our study showed that DHT upregulates renal ACE2 levels (mRNA and protein) in both diets (Figure 4A,G). However, DHT-mediated cardiac ACE2 upregulation was only observed in the presence of an obesogenic diet (Figure 3A,F). Notably, renal ACE2 expression is higher in male mice compared with female counterparts [72], a finding that may explain the DHT-mediated expression upregulation we observed in DHT-treated mice [73]. Alternatively, this increase in ACE2 could be a compensatory mechanism to counterbalance Ang II-mediated deleterious actions [74]. This notion is supported by a study showing that ACE2 is tissue-specifically upregulated in the kidney, but not the heart, of diabetic mice to exert renoprotection [75]. The interaction between androgens, ACE2, and cardiorenal injury is complex, and further studies are needed to fully understand those interactions.

In the present study, while renal TMPRSS2 mRNA levels were upregulated by DHT treatment independently of the diet, the protein expression was significantly upregulated only in HFD-DHT mice compared to their LFD-fed counterparts. This increase could be due to increased renal expression of the AR (Figure 2), which was reported to regulate TMPRSS2 transcription and translation [76,77]. This suggestion is supported by the observation that HFD-DHT mice, which showed the highest AR mRNA and protein level, also had the highest increase in TMPRSS2 (Figure 4). Moreover, correlation analysis of AR and Tmprss2 mRNAs showed a significant positive correlation (Figure 6). The regulation of TMPRSS2 by androgens has been proposed as a likely potential mechanism to explain the male predominance in the COVID-19 pandemic [78] and possibly another risk factor for severe COVID-19 in women with PCOS especially those who are overweight or obese.

Another transmembrane serine protease that we assessed in the current study was TMPRSS4, also known as channel-activating serine protease (CAP2). TMPRSS4 has been postulated as an alternative protease involved in SARS-CoV-2 viral entry into human enterocytes in the gastrointestinal tract [69] and possibly other tissues [79]. One of the main functions of TMPRSS4 is to activate the epithelial sodium channel (ENaC), which promotes sodium reabsorption in distal tubules [80]. Androgens can upregulate the α-subunit of ENaC in the kidneys and hence increase sodium reabsorption and blood pressure [81]. Interestingly, in our study, DHT-treated mice showed downregulation in renal TMPRSS4 levels (Figure 4). Additionally, TMPRSS4 mRNA expression was negatively correlated with AR mRNA (Figure 6). TMPRSS4 downregulation could be a defense mechanism to counterbalance the increased expression of ENaC channel in the kidneys. Notably, TMPRSS4 expression levels were below the detection limit in LV samples in our study, a finding consistent with human expression data [82].

There is strong clinical evidence that COVID-19 affects the heart [83]; however, cardiac Tmprss2 expression is relatively low, raising the question of the mechanism of SARS-CoV-2 mechanism of entry in such tissue. Recent studies have shown that other S-protein proteases, such as cathepsin L and furin, are highly expressed in multiple types of cardiac cells, and those could be the main proteases involved in SARS-CoV-2 entry in cardiac tissue [84]. Surprisingly, SARS-CoV-2 S protein has a furin-like cleavage site which is not present in other CoV S proteins [85]. Notably, furin is not only important for membrane-mediated viral fusion and entry, but also for the cleavage of the newly synthesized S proteins in the Golgi apparatus that proceeds the assembly and release of new virions by exocytosis [86]. In our study, cardiac and renal furin mRNA were upregulated by DHT, but furin protein was only upregulated by DHT in the kidney of LFD-fed animals (Figure 3 and Figure 4). Moreover, in the kidneys, both total and active (soluble) furin protein expression was increased by DHT (Figure 4 and Supplementary Figure S1). The increased renal furin levels in DHT-treated mice may suggest that more viral propagation is likely to occur following SARS-CoV-2 infection in PCOS probably leading to more severe COVID-19 outcomes.

Cathepsin L is an endosomal cysteine protease that mediates the cleavage of the S1 subunit of the S glycoprotein and facilitates virus and host cell endosome membrane fusion. Cathepsin L also mediates viral replication by inducing viral RNA release [87]. Ou et al. showed that SARS-CoV-2 enter cells mainly via endocytosis and that cathepsin L inhibitors blocked the viral entry in cells expressing human ACE2 [88]. While no changes in renal cathepsin L levels were observed in DHT-treated mice in LFD, it was significantly upregulated by DHT in HFD-fed mice (Figure 4K), suggesting a pathway for increased renal SARS-CoV-2 viral entry by endocytosis following infection in obese women with PCOS. Notably, despite higher cardiac cathepsin L mRNA levels in HFD-DHT mice compared to LFD-DHT mice, its protein levels were reduced (Figure 3D,I). Cathepsin L overexpression was reported to improve cardiac function, inflammation, and fibrosis in models of cardiac hypertrophy, and its deficiency in mice results in progressive dilated cardiomyopathy [89]. Thus, based on our findings, although lower viral cardiac entry via endocytosis may be expected in obese women with PCOS, they are at a higher risk of COVID-19-associated cardiomyopathy.

The main enzyme responsible for ACE2 shedding is ADAM17, a metalloproteinase enzyme that can also shed a diverse variety of membrane-anchored cell adhesion molecules, receptors, and cytokines, including TNF-α and IL-6 receptor [90]. Our findings show that ADAM17 protein expression was increased in response to DHT similarly in both diets. ADAM17 may not only facilitate the virus entry but also may enhance direct tissue damage through local TNF-α release [91]. The increased cardiac and renal ADAM17 expression in our hyperandrogenemic PCOS model suggests the possibility of local cardiac and renal injury following COVID-19 infection in the PCOS patient population. Moreover, the increased shedding of TNF-α and other cytokines could contribute to a more severe cytokine storm following SARS-CoV-2 viral infection by increasing the levels of circulating cytokines.

Circulating ACE2 levels are higher in men than in women and subjects with diabetes or cardiovascular diseases [92]. Despite similar circulating ACE2 protein levels in both vehicle and DHT-treated mice, a marked elevation in urinary ACE2 protein was observed in DHT-treated mice on both diets (Figure 5). Urinary shedding of kidney ACE2 is the main source of urinary ACE2 and is mediated by ADAM17 [93]. The increase in urinary ACE2 protein is likely due to both the DHT-mediated increase in renal ACE2 and ADAM17 protein expression (Figure 4G,L). Urinary ACE2 is increased in patients with chronic kidney disease compared with healthy controls, and a further rise in urinary ACE2 is observed in patients with diabetic nephropathy compared with patients with other renal disorders [94]. Furthermore, in adolescents with uncomplicated type 1 diabetes mellitus, urinary ACE2 protein excretion and activity levels were elevated compared with healthy controls and correlated with higher HbA1c but were not associated with eGFR, blood pressure, or albuminuria [95]. Additionally, studies in a mouse model of type 2 diabetes mellitus showed ACE2 protein is upregulated in renal tubules from diabetic mice, and the change in tubular ACE2 was translated into urinary, but not circulating, ACE2 protein increases [96]. Our findings of a remarkable increase in urinary ACE2 in DHT-treated mice irrespective of the diet may suggest that urinary ACE2 could be an early marker of renal injury in PCOS.

Correlation analysis between AR and Ace2, Tmprss2, furin, cathepsin L, and Adam17 mRNA expression showed a significant positive correlation, suggesting expression regulation by androgens through the androgen receptor (Figure 6). Those findings align with previous ones that have shown TMPRSS2 and ACE2 upregulation by androgens in other tissues. For example, TMPRSS2 protein expression is upregulated by androgens in prostate cancer cells and prostate tumors [76,77]. Moreover, ACE2 shows sexual dimorphic expression; male mice have increased renal Ace2 protein expression compared with females [72]. Furthermore, more recently, androgen signaling has been shown as a key modulator of ACE2 expression in human embryonic stem cell-derived cardiac cells [97]. Overall, our findings showed AR mRNA expression positive correlation with multiple SARS-CoV-2 viral entry proteins in the kidney, suggesting that this organ is a major target of excess androgen action in PCOS.

**Figure 7:**
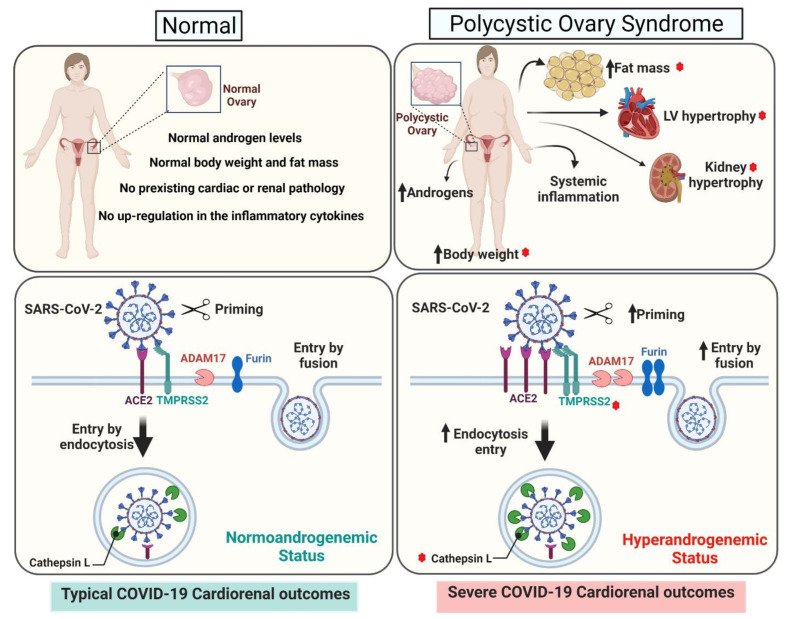
Proposed mechanisms for increased risk for severe COVID-19 cardiorenal outcomes in normal women and women with PCOS. Top panels: Women with PCOS have increased body weight, fat mass, and cardiac and renal hypertrophy. Lower panels: Androgens differentially regulate the cardiac and renal expression of SARS-CoV-2 viral entry proteins including the SARS-CoV-2 receptor ACE2 and several cellular proteases that promote viral entry and propagation. HFD exacerbates androgens-mediated obesity and cardiorenal hypertrophic response and modulates androgens’ effect on the expression of the different cellular proteases. The red stars indicates the parameters that are exacerbated with HFD.

COVID-19 is an ongoing pandemic, and little is still known about its acute and long-term effects on particular populations. Most women with PCOS present clinical or biochemical hyperandrogenism. In our study, a preclinical animal experimental model of PCOS generated by peripubertal androgen exposure showed an upregulation of cardiac and renal SARS-CoV-2 viral entry proteins, an effect differentially modulated by an obesogenic diet. In particular, the kidney showed the most striking upregulation by excess androgens of the SARS-CoV-2 receptor ACE2 as well as multiple cellular proteases (TMPRSS2, furin, cathepsin L, and ADAM17) involved in viral entry. HFD had a modulatory effect on several SARS-CoV-2 viral entry proteins; in particular, in the heart DHT upregulated SARS-CoV-2 receptor ACE2 mRNA and protein only in the animals maintained in an obesogenic diet. An exciting finding was that androgens not only upregulated their own receptor, the AR, but also that HFD by itself upregulated renal AR protein expression. In summary, our study suggests that women with PCOS with hyperandrogenism may be at a higher risk of worsened cardiac and renal outcomes when suffering from COVID-19. Moreover, our findings on cardiac and renal AR and SARS-CoV-2 viral entry proteins regulation indicate possible molecular mechanisms by which excess androgens and HFD interact and may lead to increased cardiorenal COVID-19 severity in PCOS (Figure 7). Collectively, our study highlights the strong need for effective, selective, and safe androgen receptor blockers to mitigate not only metabolic and cardiovascular symptoms in women with PCOS but also possible COVID-19-associated outcomes. Our study also shows that weight loss in PCOS not only has reproductive and cardiometabolic benefits but could also have a protective effect on COVID-19-associated outcomes. Finally, our findings on AR and SARS-CoV-2 viral entry proteins may apply to other populations with endogenous or exogenous elevated androgens.

## 4. Materials and Methods

### 4.1. Animals

Three-week old female C57BL/6NCrl mice were obtained from Charles Rivers (Wilmington, MA, USA). Animals were maintained under standard housing conditions under controlled temperature and humidity environment with a 12:12 h light-dark cycle. The mice were maintained in either high-fat diet (HFD, 60% kcal fat) (D12492, Research Diets, Inc., New Brunswick, NJ, USA) or control low-fat diet (LFD, 10% kcal fat, matched for sucrose content, Research Diets, Inc., D12450J). Food and water were provided *ad libitum* throughout the study. All experimental protocols were performed in accordance with the National Institutes of Health’s Guide for the Care and Use of Laboratory Animal 8th edition (2011) and reviewed and approved by the Institutional Animal Care and Use Committee of the University of Mississippi Medical Center.

### 4.2. Experimental Design

Following a four-day acclimatization period, the peripubertal androgen exposure model of PCOS was induced in mice following the method of Caldwell, et al., [98] with minor modification, Briefly, mice were randomly assigned to be implanted s.c. with dihydrotestosterone (DHT, A2570–000, 8 mg/tube, Steraloids Inc., Newport, RI, USA) in Silastic tubes (length: 1.5 cm; id: 1.47 mm; od: 1.95 mm, catalog 508–006, Dow Corning Corp, Midland, MI, USA) or empty (vehicle) Silastic tubes (*n* = 16/group). Both, DHT-treated and vehicle animals, were randomly assigned to be maintained in HFD or LFD (*n* = 8/group) for 90 days. At the end of the experimental period, the blood was collected via cardiac puncture, followed by perfusion with saline under isoflurane gas anesthesia. The kidneys and LV were harvested, weighted, and flash frozen in liquid nitrogen. Tissues were stored at −80 °C for further processing. Tissue weights were corrected by tibia length (TL) measured with an analytical caliper. At the end of the experiment, all DHT-filled Silastic tubes were removed and checked for the presence of remnant DHT confirming the continuous delivery of DHT through the experimental period.

### 4.3. Body Composition

Weekly body weights were recorded throughout the experimental period. Additionally, weekly body composition (lean and fat mass) measurements were performed by EchoMRI (4in1-EF-016 model Body Composition Analyzer; EchoMRI, Houston, TX, USA).

### 4.4. mRNA Expression Quantification

Total kidney and LV RNA were extracted, DNAse treated, quantified, and reverse transcribed (5 µg), as previously described [37,99]. Quantitative RT-PCR was performed using TaqMan gene expression assays (Applied Biosystems, Foster City, CA, USA) for Ace2 (Mm01159006_m1), Tmprss2 (Mm00443687_m1), Tmprss4 (Mm00520486_m1), Furin (Mm00440646_m1), Ctsl (Mm00515597_m1), Adam17 (Mm00456428_m1), Gapdh (Mm99999915_g1), B2m (Mm00437762_m1), Actb (Mm02619580_g1), and 18S rRNA (Hs99999901_s1). Reactions were performed with Luna universal probe qPCR master mix (New England Biolabs, Ipswich, MA, USA). Reactions were cycled (50 °C for 2 min, 95 °C for 20 s, followed by 40 cycles of 95 °C for 1 s and 60 °C for 20 s) in a QuantStudio 3 (Applied Biosystems) cycler. PCR product quantification was performed by the ∆∆Ct quantification method and expressed as arbitrary units (AU) standardized against the geometric mean of four reference genes (Actb, B2m, Gapdh, and 18S rRNA).

### 4.5. Western-Blot Analysis

Kidney and LV tissues were homogenized in radioimmunoprecipitation assay (RIPA) buffer containing Halt protease and phosphatase inhibitor cocktail (ThermoFisher Scientific, Waltham, MA, USA). The total protein concentration was quantified with the bicinchoninic acid protein assay kit (ThermoFisher Scientific, Waltham, MA, USA). Fifty micrograms of total protein were resolved by SDS-PAGE and transferred to PVDF membranes. The membranes were blocked with 5% nonfat dry milk in Tris-buffered saline containing 0.1% Tween 20 for 1 h at room temperature, and then incubated overnight at 4 °C with the following primary antibodies: Androgen receptor (1:2000 for the LV, 1:1000 for the kidney; Abcam ab133273, Cambridge, MA, USA), ACE2 (1:10,000; Abcam ab108252, Cambridge, MA, USA), TMPRSS2 (1:1000 for the LV, 1:10,000 for the kidney; Abcam ab92323, Cambridge, MA, USA), TMPRSS4 (1:5000; Proteintech 11283–1-AP, Rosemont, IL, USA), furin (1:5000; Abcam ab183495, Cambridge, MA, USA), cathepsin L (1:500; ThermoFisher Scientific MA5–23891, Waltham, MA, USA), ADAM17 (1:500; ThermoFisher Scientific PA5-27395, Waltham, MA, USA) or GAPDH (1:3,000,000; Cell Signaling Technology 5174, Danvers, MA, USA). The membranes were then incubated with horseradish peroxidase-conjugated goat anti-rabbit or anti-rat IgG secondary antibodies (1:10,000; Jackson ImmunoResearch Laboratories 111-035-003 and 112-035-003, West Grove, PA, USA) for 1 h at room temperature. Detection by chemiluminescence was performed with SuperSignal West Pico PLUS kit (ThermoFisher Scientific, Waltham, MA, USA). The ChemiDoc MP imaging system (Bio-Rad, Hercules, CA, USA) and ImageJ (National Institutes of Health, Bethesda, MD, USA) were used to capture and quantify the images, respectively.

### 4.6. Serum and Urinary ACE2

Serum was obtained from blood after one-hour incubation at room temperature and centrifugation at 1300× *g* for 10 min at 4 °C. Urine was collected from animals placed in 24-h metabolic cages after 12 weeks of treatment. ACE2 protein levels were quantified in serum and urine samples using a commercial ACE2 ELISA kit (Abcam ab213843, Cambridge, MA, USA) according to the manufacturer’s instructions. Urinary ACE2 was corrected for urinary creatinine, which was measured using the Mouse Creatinine Colorimetric Kit (Crystal Chem 80350, Elk Grove Village, IL, USA).

### 4.7. Statistical Analysis

The results are presented as mean  ±  SEM. Statistical analysis were performed using two-way ANOVA followed by Fisher’s LSD *post hoc* tests. Statistical calculations were performed with Prism software (GraphPad, Inc., San Diego, CA, USA, version 8.4.3). Differences between groups were considered significant if *p* ≤ 0.05. The number of animals required per group was calculated by performing a power analysis using Statmate software (version 2.0, GraphPad, Inc.). A sample size of 4–8 animals/group has 80–99% power to detect a difference equivalent to 0.7-fold of control mean values between groups at a significance level (alpha) of 0.05 (two-tailed).

## Data Availability

Data is contained within the article or supplementary material.

## Supplementary Materials

Supplementary materials can be found at https://www.mdpi.com/article/10.3390/ijms22189746/s1.
