# Supplementary material for: Cardiac and Renal SARS-CoV-2 Viral Entry Protein Regulation by Androgens and Diet: Implications for Polycystic Ovary Syndrome and COVID-19
Source: Int J Mol Sci. 2021 Sep 9;22(18):9746. doi: 10.3390/ijms22189746 (PMC8470275; doi:10.3390/ijms22189746)
Supplement: Supplementary file 1 [file ijms-22-09746-s001.zip › ijms-1356907-supplementary.pdf]

Supplemental Figure S1

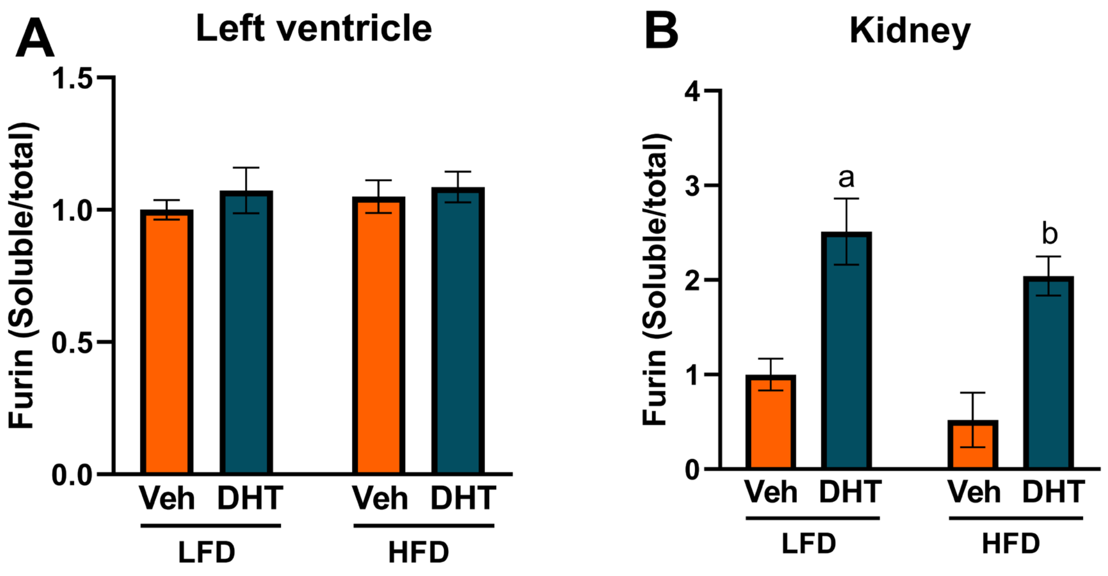

Supplemental Figure S1: Effect of DHT and diet on renal soluble furin protein expression. Animals were treated with dihydrotestosterone (DHT) or vehicle (Veh) and maintained in low (LFD) or high (HFD) fat diet for 90 days. Left ventricle (A) and kidney (B) soluble and total furin protein were quantified by Western-blot (N = 4/group). Data are expressed as mean ± SEM. Data were analyzed by two-way ANOVA followed by Fisher's LSD test. [a]$P < 0.05$ vs. LFD-Veh; [b]$P < 0.05$ vs. LFD-Veh.
